# Water outage increases the risk of gastroenteritis and eyes and skin diseases

**DOI:** 10.1186/1471-2458-11-726

**Published:** 2011-09-25

**Authors:** Ling-Ya Huang, Yu-Chun Wang, Chung-Ming Liu, Trong-Neng Wu, Chang-Hung Chou, Fung-Chang Sung, Chin-Ching Wu

**Affiliations:** 1 Department of Public Health, China Medical University, Taichung 404, Taiwan; 2 Department of Bioenvironmental Engineering, Chung Yuan Christian University, College of Engineering, Chung Li 320, Taiwan; 3 Department of Atmospheric Sciences, National Taiwan University, Taipei 106, Taiwan; 4 Institute of Ecology and Evolutionary Biology, College of Life Science, China Medical University, Taichung 404, Taiwan; 5 Institute of Environmental Health, National Taiwan University, Taipei 100, Taiwan

## Abstract

**Background:**

The present study used insurance claims data to investigate infections associated with short-term water outage because of constructions or pipe breaks.

**Methods:**

The present study used medical claims of one million insured persons for 2004-2006. We estimated incidences of gastroenteritis and eye and skin complaints for 10 days before, during, and after 10 days of water supply restriction for outpatient visits and for emergency and in-patient care combined.

**Results:**

There was an increase in medical services for these complaints in outpatient visits because of water outages. Poisson regression analyses showed that increased risks of medical services were significant for gastroenteritis (relative risk [RR] 1.31, 95% confidence interval [CI] 1.26-1.37), skin disease (RR 1.36, 95% CI 1.30-1.42), and eye disease patients (RR 1.34, 95% CI 1.26-1.44). Similar risks were observed during 10-day lag periods. Compared with those in cool days, risks of medical services are higher when average daily temperature is above 30°C for gastroenteritis (RR 12.1, 95% CI 6.17-23.7), skin diseases (RR 4.48, 95% CI 2.29-8.78), and eye diseases (RR 40.3, 95% CI 7.23-224).

**Conclusion:**

We suggest promoting personal hygiene education during water supply shortages, particularly during the warm months.

## Background

Water supply is essential to the maintenance of proper sanitation and personal hygiene. Inadequate water supply increases the risks of diarrhea, other infectious diseases, and related complaints. Natural disasters, such as floods or droughts, contribute to inadequate water supply and may lead to the outbreak of water-borne and water-washed diseases, including gastroenteritis and respiratory tract and skin infections [1-7.] Huge diarrhea outbreaks cause cholera and *Escherichia coli *infections after a flood [8,9]. The diarrhea outbreak in Milwaukee, which was caused by cryptosporidium oocysts and which affected 403,000 victims, was caused by a filtration system that malfunctioned after a heavy storm [10-12]. The risk of waterborne gastrointestinal illness also has been associated with drinking water turbidity under current water treatment practices even without heavy precipitation [13,14].

Water outage is another public and personal hygiene concern because it means the lack of water for use in laundry, kitchen, and restroom maintenance. Water outage following a power outage has always been a major problem in communities and in hospitals. Inadequate water services for home usage increased the risks of pneumonia and influenza infections among families in rural Alaska [15]. Surprisingly, information on diseases related to water supply shortage has not been well investigated and reported in short-term water outages in communities. This is certainly a drawback in the prevention of diseases because of the lack of water.

Taiwan is an island located in the western part of the Pacific Ocean that frequently receives undistributed rainfall. In fact, water treatment plants may shut down if water turbidity resulting from heavy precipitation during typhoons is too high to treat. Water outage may also occur for other reasons, such as during constructions or when water supply pipes are broken. No information has been reported on health effects during water outage intervals. Therefore, we used water supply records, weather monitoring data, and national insurance claims to assess whether or not water outages caused by constructions or broken water supply pipes lead to increased incidences of infectious diseases. We placed particular focus on gastroenteritis and skin and eye complaints.

## Methods

### Environmental data

Water outage data for the study period were provided by the Taiwan Water Corporation. These data were used to identify affected areas, dates of occurrences, duration, and reasons for outages, such as broken pipes or constructions.

Considering the effects of weather on infectious diseases, we used daily weather measurements and events data from the Central Weather Bureau (CWB). In the present study, typhoons were defined based on typhoon warnings released by the CWB, which contained information on time, duration, and areas hit by each typhoon that appeared during the summer months. We also used daily average temperatures to evaluate the relationship between infections and temperatures.

Aside from typhoon data, flood data from the National Fire Agency, as well as data on affected areas, dates of occurrences, and receding dates from the Ministry of Interior Affairs, were also used.

To clarify further the association between contamination of drinking water and water outage, we also obtained drinking water quality inspection data from Taiwan's Environmental Protection Administration's (EPA) Executive Yuan for data analyses. Data on the most probable number of coliform tests in water samples were used.

### Health insurance data

These insurance claims were randomly selected from the National Health Insurance database, which was established and provided by the National Health Research Institute (NHRI). The reported coverage rate among the population of 23 million has been 96% or higher since 1996 [7]. The medical claims data provided information on scrambled identifications, gender, birthdays, types of services and diagnoses, dates of admissions and discharges, and medical institutions providing services for each patient. Eight cities and counties were chosen to represent urban and suburban areas for northern (Taipei and Taoyuan), central (Taichung and Changhua), southern (Kaohsiung and Tainan), and eastern (Taitung and Hualien) Taiwan. The present study used medical claims of one million insured persons and environmental data for 2004-2006. The total study population size covered in the insurance system in these areas in 2005 was 624,176. The health insurance data used in this study obtained from the NHRI have been safeguarded for the privacy and confidentiality with scrambled identifications. This study was thus exempted from ethical review.

We retrieved medical records before, during, and after each water outage for medical services for gastroenteritis, eye and skin infections, and other complaints, in accordance with the *International Classification of Diseases, 9th Revision, Clinical Modification *(ICD-9 CM). The usage of medical services in the present study includes outpatient visits, and emergency and inpatient cares combined (EICC). The ICD-9-CM codes representing gastroenteritis were ICD-9 CM 001-009, 535, 5362, 555, 5582, 5589, 567, 5689, 578, 787, and 789. Infectious skin diseases (ICD-9 CM 680-686), acariasis (ICD-9 CM 133), mycoses of skin (ICD-9 CM 110 and 111), and rashes (ICD-9 CM 7821) were selected from a group of skin diseases. Meanwhile, codes for eye diseases were conjunctivitis (ICD-9 CM 3720), inflammation of the eyelids (ICD-9 CM 3734, 3735, and 3736), and trachoma (ICD-9 CM 076).

### Data analysis

Considering that the effects of water outages could be delayed for a few days, we divided each water outage event into three periods, namely, 10 days with normal water supply before any water outage (normal period), the actual day(s) with water outages ( lag 0), and 10 days after the outages (10-day lag). To exclude effects from typhoons and floods, water outages during these weather events were excluded.

We calculated the incidence rates of medical services, including outpatient visits and EICC for gastroenteritis and eye and skin complaints in person-days in normal, lag 0, and 10-day lag periods. Both the outage period rate to the normal water supply period rate ratio and the 10-day lag period rate to the normal water supply period rate ratio were calculated separately for each type of infection.

We performed the Poisson regression model to measure the risks of medical visits associated with water outages. Afterward, we estimated the average daily temperature (<15, 15-20, 20-24, 25-29, and 30+°C) specific outage-to-normal relative risks (RR), and 95% confidence interval (CI) for selected diseases. Adjustment was made for calendar year, month, holiday, geographic area, sex, age, gross domestic product (GDP) index, and education index. The multivariate-adjusted models, which served as a control for the daily average temperature instead of age (<15, 15-64, and 65+ years), were repeated to estimate the age-specific outage to normal-period RR. The model is specified as follows:

Log (∆Yi) = α + β_1_X_1_ + ... + β_p_X^p^.

RRs of daily area-disease-specific outpatient visits and EICC (ΔYi) associated with water outage period and a 10-day lag period, compared with the normal period, were estimated after controlling for the covariates, such as area, sex, age (<15, 1-64, and 65+ years), daily average temperature (<15, 15-20, 20-24, 25-29, and 30+°C), daily relative humidity, GDP index, education index, year, and month. RR and 95% CI were calculated based on the exponential transformation of βi estimations.

To clarify whether water outage-related diseases resulted from water-borne or water-washed pathways, we compared the water supply quality during the three observed periods of the water outages. Based on test results, Taiwan EPA recorded each tested drinking water sample as either "unqualified" or "qualified." Matching with the sampling time, we calculated the period-specific "water unqualified rate (%)" by dividing the number of unqualified water samples by the total number of tested water samples. The Chi-square test was used to check if the water test frequency differs with periods. The association between unqualified rate (%) and water outage period (normal period, lag 0, and 10-days lag) was evaluated using generalized linear models. All statistical analyses were performed using SAS version 9.1 (SAS Institute Inc., Cary, NC, USA).

## Results

Public water supply rates of the areas studied ranged from 77.05%-99.59% (average of 92.73%). In all, there were 193 island-wide water outages in 2004-2006, and 168 events (112 caused by broken pipes and 56 caused by constructions) occurred in the study areas. The average duration of water outages was 15.7 (95% CI 6.7-36.3) hours for these areas.

Outpatient visit rates during water outages were elevated for gastroenteritis (63.9 per 100,000 person-days), and those for the 10-day lag period (60.9 per 100,000 person-days) were higher than the rates during normal supply periods (57.5 per 100,000 person-days) with significant rate ratios (Table 1). No significant changes were observed for EICC. Similar patterns were observed for skin and eye diseases.

**Table 1:** Incidences of outpatient and EICC services for gastroenteritis and skin and eyes diseases associated with water outage and rate ratios to normal period in 2004-2006 in Taiwan

		**Normal period **^**a**^	Outage period	10-day lag period
	Person-days	77563341	21479559	127184866
**Gastroenteritis**				
Outpatient	N	44590	13732	77399
	Rate ^b^	57.5	63.9	60.9
	Ratio	1.0	1.11(1.09-1.13)***	1.06 (1.05-1.07)***
				
EICC^c^	N	2021	546	3404
	Rate ^b^	2.61	2.54	2.68
	Ratio	1.0	0.98 (0.89-1.07)	1.03 (0.97-1.09)
				
**Skin diseases**				
Outpatient	N	39113	12580	68181
	Rate ^b^	50.4	58.6	53.6
	Ratio	1.0	1.16(1.14-1.18)***	1.06 (1.05-1.08)***
				
EICC^c^	N	394	120	628
	Rate ^b^	0.51	0.56	0.49
	Ratio	1.0	1.10 (0.90-1.35)	0.97 (0.86-1.10)
				
**Eye diseases**				
Outpatient	N	9163	2980	17102
	Rate ^b^	11.8	13.9	13.4
	Ratio	1.0	1.17(1.13-1.22)***	1.14 (1.11-1.17)***
				
EICC^c^	N	27	7	52
	Rate ^b^	0.03	0.03	0.04
	Ratio	1.0	0.94 (0.41-2.15)	1.17 (0.74-1.87)

^a^Water outages because of typhoons and floods were excluded.

^b^1/100,000 person-days by period

^c^Emergency room visits and inpatient visits combined

Poisson regression analysis results also showed that the overall relative risks during water outages were 1.31 (95% CI 1.26-1.37) for gastroenteritis, 1.36 (95% CI 1.30-1.42) for skin diseases, and 1.34 (95% CI 1.26-1.44) for eye diseases after controlling for daily average temperature and relative humidity, year, month, area, sex, age, GDP index, and education index (Table 2). All these RRs continued until the 10-day lag periods for these infections.

**Table 2:** Multivariate Poisson regression analysis measuring relative risks for gastroenteritis, and infections of skin and eyes associated with water outage periods, and the daily average temperature after controlling for potential confounders in Taiwan, 2004-2006

	Gastroenteritis	Skin disease	Eye disease
Period	RR (95% CI)	RR (95% CI)	RR (95% CI)
**Outage**	1.31 (1.26-1.37)***	1.36 (1.30-1.42)***	1.34 (1.26-1.44)***
**10-day lag**	1.26 (1.23-1.30)***	1.30 (1.26-1.33)***	1.35 (1.30-1.41)***
**Normal**	1.0	1.0	1.0
p value ^a^	<0.0001	<0.0001	<0.0001

* p < 0.05; ** p < 0.001; *** p < 0.0001

※ Model includes water outage period, daily average temperature, daily relative humidity, year, month, holiday, area, sex, age, GDP index, and education index.

a: LR statistics for type 3 analysis

Analysis of temperature-specific outage-related RRs demonstrated that risks tend to be higher during hot weather (Table 3). As the ambient temperature increased to over 30°C, RRs during water outages were 12.1 (95% CI 6.17-23.7) for gastroenteritis, 4.48 (95% CI 2.29-8.78) for skin diseases, and 40.3 (95% CI 7.23-224) for eye diseases. High RRs for these three types of infection remained in the 10-day lag period as the ambient temperature increased to over 30°C. Results of other covariates showed that RRs of medical services for all three sites of diseases ranged from 1.23-1.50. Infection risk associated with high temperature was much stronger than that associated with the other covariates.

**Table 3:** Covariates associated relative risks of gastroenteritis, skin infections, and eye diseases during water outage compared with those during normal period in Taiwan, 2004-2006

		Outage period (vs. normal period)	10-day lag period (vs. normal period )
		RR (95% CI)	RR (95% CI)
**Gastroenteritis**			
**Temperature (°C)**	**< 15**	1.19(1.05-1.34)*	1.15(1.08-1.23)***
	15-19	1.19(1.10-1.28)***	1.10(1.05-1.16)***
	**20-24**	1.20(1.12-1.28)***	1.24(1.19-1.29)***
	**25-29**	1.69(1.56-1.83)***	1.61(1.53-1.71)***
	30+	12.1(6.17-23.7)***	11.3(6.10-20.8)***
	p for trend	0.16	0.16
			
**Humidity (%)**	**< 67**	1.31(1.24-1.37)***	1.28(1.24-1.32)***
	**67-83**	1.36(1.23-1.51)***	1.21(1.13-1.29)***
	**84+**	1.25(1.11-1.41)**	1.29(1.21-1.38)***
	**p for trend**	0.63	0.93
			
**Age (years)**	**< 15**	1.30(1.21-1.40)***	1.23(1.17-1.28)***
	15-64	1.32(1.24-1.41)***	1.28(1.23-1.33)***
	**65+**	1.29(1.18-1.40)***	1.26(1.20-1.33)***
	**p for trend**	0.79	0.59
			
**Education Index**	**0.82-0.84**	1.38(1.29-1.49)***	1.39(1.33-1.46)***
	**0.85+**	1.30(1.23-1.36)***	1.23(1.19-1.26)***
	**P value**	0.97	0.91
			
**Skin infections**			
**Temperature(°C)**	**< 15**	1.14(0.99-1.30)	1.10(1.02-1.18)*
	15-19	1.26(1.16-1.37)***	1.23(1.17-1.30)***
	**20-24**	1.26(1.17-1.35)***	1.26(1.20-1.31)***
	**25-29**	1.63(1.50-1.77)***	1.52(1.44-1.61)***
	30+	4.48(2.29-8.78)***	5.19(2.94-9.17)***
	**p for trend**	0.12	0.13
			
**Humidity (%)**	**< 67**	1.38(1.31-1.45)***	1.33(1.29-1.38)***
	**67-83**	1.31(1.18-1.46)***	1.15(1.07-1.24)***
	**84+**	1.30(1.14-1.49)***	1.38(1.28-1.49)***
	**p for trend**	0.26	0.87
			
**Age (years)**	**< 15**	1.43(1.30-1.58)***	1.27(1.19-1.35)***
	15-64	1.35(1.26-1.44)***	1.30(1.25-1.35)***
	**65+**	1.36(1.24-1.48)***	1.30(1.23-1.38)***
	**p for trend**	0.41	0.33
			
**Education Index**	**0.82-0.84**	1.42(1.31-1.54)***	1.44(1.36-1.52)***
	**0.85+**	1.35(1.28-1.42)***	1.26(1.23-1.31)***
	**p value**	0.96	0.90
			
**Eye infections**			
**Temperature(°C)**	**< 15**	1.05(0.81-1.36)	1.14(1.004-1.30)*
	15-19	1.28(1.10-1.48)*	1.27(1.15-1.39)***
	**20-24**	1.10(0.98-1.24)	1.24(1.16-1.32)***
	**25-29**	1.73(1.54-1.94)***	1.65(1.52-1.79)***
	30+	40.3(7.23-224)***	18.0(3.56-90.7)**
	**p for trend**	0.17	0.17
			
**Humidity (%)**	**< 67**	1.33(1.22-1.44)***	1.39(1.32-1.46)***
	**67-83**	1.27(1.07-1.50)*	1.20(1.08-1.34)**
	**84+**	1.50(1.20-1.86)**	1.35(1.18-1.54)***
	**p for trend**	0.50	0.87
			
**Age (years)**	**< 15**	1.34(1.17-1.53)***	1.38(1.27-1.50)***
	15-64	1.36(1.24-1.49)***	1.32(1.24-1.39)***
	**65+**	1.27(1.07-1.51)*	1.48(1.33-1.64)***
	**p for trend**	0.47	0.58
			
**Education Index**	**0.82-0.84**	1.41(1.23-1.61)***	1.53(1.39-1.67)***
	**0.85+**	1.31(1.21-1.42)***	1.28(1.22-1.34)***
	**p for trend**	0.95	0.86

* p < 0.05; ** p < 0.001; *** p < 0.0001

※Model included water outage period, daily average temperature, daily relative humidity, year, month, holiday, area, sex, age, GDP index, and education index were included in the model.

Table 4 shows the water quality test results of the public water supply by water outage status. Unqualified rates of water quality test were slightly higher during water outages and 10-day lag periods (0.7% vs. 0.2%). However, there were no statistically significant differences observed for water quality tests and most probable number of coliform tests among the three periods.

**Table 4:** Water quality test and most probable number test of water samples associated with water outage in Taiwan, 2004-2006

		Normal period	Outage period	10-day lag period	p value
		**n (%)**	**n (%)**	**n (%)**	

**Water quality test (day)**	Yes	339 (22.2)	94 (25.8)	456 (22.1)	0.29
	No	1186 (77.8)	271 (74.2)	1606 (77.9)	
	Unqualified rate (%)	0.18	0.69	0.66	0.31
					
**MPN test (day)**	Yes	168 (11.0)	45 (12.3)	195 (9.46)	0.13
	No	1357 (89.0)	320 (87.7)	1867 (90.5)	
	Unqualified rate (%)	0.03	0	0	0.38
	mean ± sd.	2.84 (9.84)	2.16 (5.08)	2.54 (8.82)	0.47

MPN: most probable number of coliform test result.

## Discussion

To the best of our knowledge, this is the first study that provides information on gastroenteritis and on complaints related to skin and eye diseases during and after water outages for the population of a subtropical island. We found that incidences of gastroenteritis, skin diseases, and eye diseases among outpatient visits are higher during water outage periods than during normal periods. Medical services are availed of relatively more for gastroenteritis and skin infections than for eye infections, including both outpatient visits and measures of EICC.

Water outages were usually short, with an average of 15.7 (95% CI 6.7-36.3) hours. However, they still caused a significant effect on personal hygiene in the communities affected. Our observations revealed that medical service utilization for these diseases during water outages was approximately elevated from 23% to 48% among age groups compared with that during normal water supply periods.

Several studies have reported an association between heavy precipitation and water-borne diseases [1,10-12,16]. Although the people in Taiwan have experienced many floods before, they have not yet experienced a cryptosporidiosis outbreak, such as that which occurred in Milwaukee [12]. This difference may have to do with water utilization behavior, given that the people in Taiwan are used to drinking boiled water. Boiling contaminated water during water outages makes it safe to drink. However, people may still become infected when unboiled contaminated water containing microorganisms come into contact with food, the eyes and cuts on the skin. People may have to wash less often and because of water scarcity. Coincidentally, inadequate personal hygiene practices could increase potential risks for gastrointestinal disorders, and skin and eye infections or allergies. In the present study, we clarified this pathway of infection by analyzing the water supply quality reported during the study periods. No significant differences were found. Hence, we believe that the lack of water access is the main cause of increased occurrences of infectious diseases associated with water shortages in Taiwan.

The present study further examined whether risks of infections were associated with water quality. Water quality test results showed that the unqualified rates were alike during water outages and lag periods, but were slightly higher than the normal period (0.7% vs. 0.2%, *p *= 0.31). Although this difference is not statistically significant, the effects of outages still lead to higher infection risks. Turbidity threshold for action, if any, should perhaps be questioned.

In the present study, we also found that the risks of skin and eye diseases, and gastroenteritis increases as temperature increases during water outages (Table 3). This demonstrates a dose-response relationship between increased contact with microorganisms and temperatures, i.e., when it is warmer, there is greater risk of contracting infections. Pathogens are generally associated with temperature [17]. The effects of temperature on gastroenteritis vary among countries and between seasons because temperature acts on both medical search and exposure routes. Checkley et al. found that, in Peruvian children, the RR of daily hospital admissions for diarrheal diseases ranges from 1.04-1.12 as ambient temperature increases to 1°C. Hashizume et al. also found a linear association between daily non-cholera diarrhea and average temperature increase in Bangladesh [18]. Even the seasonality of enterovirus isolation is observed in both clinical cases [19,20] and environmental water samples, which are higher during warmer months [19-23]. The weather in Norway is much colder than in Taiwan. Nygård et al. found that households exposed to breaks and maintenance works in the water distribution system are 1.58-fold more likely to suffer from gastrointestinal illnesses than unexposed households [24].

Aside from public water supply, the usage of underground and well water may increase risks of infections. For instance, the outbreak of *E. coli *O157:H7 in New York in 1999 was linked to contaminated well water [23]. Curriero et al. reviewed 548 water-borne disease outbreaks and found that 36% of such outbreaks were caused by underground water contamination associated with extreme precipitation [1]. However, approximately only 8.4% of the population in Taiwan used water sources other than the public water supply, and most of them resided in suburban areas (3.8%). This was a minor part in our study areas. Thus, we were unable to determine whether people using underground or well water suffered from increased infection risks as well. The other limitation of our study is the fact that some patients may not seek health care and, thus, may not file insurance claims. However, the underestimated case pattern should not be significantly different between normal water supply days and outage days.

The strength of the present study is the fact that the daily data of both water supply records and weather monitoring results were available and can be linked to the reimbursement claims data on health insurance. The claims data reduced self-reported bias, making it possible to observe individuals who were more likely affected by water outages. With daily average temperatures linked to incidences of diseases, observing the effects of weather on the relationship between water outages and the use of medical services is also possible. To the best of our knowledge, this is the first study that provides a link to the aforementioned data sets.

## Conclusions

The present study demonstrates that a short water outage may increase risks of gastroenteritis and infections of the eyes and skin. These results also demonstrate that infection prevention measures should be heightened during warm days. In Taiwan, almost all buildings and households are equipped with water storage facilities. Water supply companies should inform the community in advance before any water-related construction is conducted and urge residents to fill their storage facilities with enough water. In case of broken pipes, water supply companies are obliged to traffic enough water to an affected community. Residents should be informed in advance as to where they can procure safe water for drinking, cooking, and personal hygiene use, including bottled, boiled, or treated water. At the same time, the local health department should make specific recommendations for boiling or treating water in areas affected by water outages.

## Competing interests

The authors declare that they have no competing interests.

## Authors' contributions

All authors have been involved in this study. LYH, YCW, CML, TNW, CHC, and FCS have designed and obtained funds. LYH and YCW analyzed data. LYH, YCW, and CCW drafted the manuscript. FCS and CCW finalized the manuscript. All authors have read and approved the final version of the manuscript.

## Pre-publication history

The pre-publication history for this paper can be accessed here:

http://www.biomedcentral.com/1471-2458/11/726/prepub
